# A Two-Pouud Child

**Published:** 1886-06

**Authors:** S. L. Post

**Affiliations:** Alvarado, Johnson County, Texas


### A TWO POUND CHILD.

*By S. L. Post, M. D., Alvarado, Johnson County, Texas.*

### REGULAR MENSTRUATION DURING GESTATION.

For Daniel's Texas Medical Journal.

MRS. L. age about twenty-three years, and mother of three children. On the 14th day of May, 1886, I was called to Mrs. L. and found her in active labor. Soon after my arrival I delivered her of a two pound fœtus at full term. As usual I examined for pulsations in the umbilical cord but to my surprise I found none, and after further examination I discovered that the cord was detached from placenta at point of union to placenta, cord being cold, and in an atrophied state. After diligent work with the infant I succeeded in getting it to breathe, and since its birth is doing as well as any infant that I have delivered under more favorable circumstances. The husband of Mrs. L. informs me that during her entire gestation she menstruated regularly, and for the last four weeks has flooded profusely; unable to attend to her domestic affairs.

Now the question is could not the fœtus live in uterus with cord detached from placenta? One of my learned colleagues says it is utterly impossible for life to be sustained any length of time, after separation of cord from placenta, during uterine life. Now, I do not pretend to say how long the cord was separated from placenta before delivery. After placing the infant in the hands of nurse, I found by digital examination that the placenta was adherent to fundus of womb; and by manipulating womb with hand introduced into vagina, I succeeded in removing placenta. All of my brother physicians in the town of Alvarado that I have talked with about the case, say that they have never seen any thing of the kind; one of them a prominent practitioner of thirty years standing. I am well aware of the fact that the fœtus receives its nourishment from the mother through the umbilical cord—but the question which arises in my mind, is could not the child probably live a few hours, or even a few days, with the cord separated from the placenta? The theory of fasting is advocated by some of our learned men, that an individual can live without food for a considerable period—and the question is, can a fœtus live with nourishment cut off;or in other words are they so constituted that they cannot fast for the shortest period? I write this article in justice to the case, and to have the opinion of yourself and other learned medical men on the subject.

